# Impact of a Mediterranean diet on prevention and management of urologic diseases

**DOI:** 10.1186/s12894-024-01432-9

**Published:** 2024-02-26

**Authors:** Mark I Sultan, Shady A Ibrahim, Ramy F Youssef

**Affiliations:** Department of Urology, University of California, 3800 Chapman Ave, Suite 7200, Irvine: Orange, CA 92868 USA

**Keywords:** Lifestyle intervention, Mediterranean diet, Sexual dysfunction, Urinary symptoms, Stone disease, Urologic cancers

## Abstract

Compared to a Western diet, the Mediterranean diet moves away from red meat and processed foods. Universally regarded as a healthier dietary alternative, the Mediterranean diet has garnered scientific endorsement for its ability to confer an array of compelling benefits. These health benefits encompass not only a lowered incidence of Type 2 diabetes with a reduction in obesity, but also a robust protective effect on cardiovascular health. Extensive literature exists to corroborate these health benefits; however, the impact of a Mediterranean diet on urologic diseases, specifically sexual dysfunction, lower urinary tract symptoms, stone disease, and urologic cancers are not well studied. Understanding how dietary habits may impact these urologic conditions can contribute to improved prevention and treatment strategies.

A total of 955 papers from PubMed and Embase were systematically reviewed and screened. After exclusion of disqualified and duplicated studies, 58 studies consisting of randomized controlled trials, cohort studies, cross sectional studies, reviews and other meta-analyses were included in this review. 11 primary studies were related to the impact of a Mediterranean diet on sexual dysfunction, 9 primary studies regarding urinary symptoms, 8 primary studies regarding stone disease, and 9 primary studies regarding urologic cancers. All primary studies included were considered of good quality based on a New-Castle Ottawa scale. The results demonstrate a Mediterranean diet as an effective means to prevent as well as improve erectile dysfunction, nephrolithiasis, lower urinary tract symptoms, and urinary incontinence. The review highlights the need for additional research to study the impact of diet on urologic cancers and other urologic conditions such as premature ejaculation, loss of libido, female sexual dysfunction, and overactive bladder.

**Supplementary Information:**

The online version contains supplementary material available at 10.1186/s12894-024-01432-9.

## Introduction

The Mediterranean Diet (MD), rooted in the dietary habits of the Mediterranean basin, reflects the nutritional practices of the region’s native population [1]. The diet consists largely of whole grains and cereals such as oats and barley, legumes such as beans and lentils, vegetables, fruits, and nuts such as walnuts and pine nuts [2, 3]. Furthermore, the diet’s hallmark feature is its substantial reliance on olive oil, particularly extra virgin olive oil, known for its abundance in monounsaturated fats and minimal saturated fat content [2, 3]. Overall, the MD largely avoids the consumption of dairy and red meat while focusing more on fish and poultry as a source of protein [3]. Nutritionally, the diet’s total daily energy intake is roughly 40–50% carbohydrates, 15–20% proteins, and 30–40% fats [4].

By comparison, the typical Western diet is characterized by the intake of red meats, sweets, and processed foods, resulting in lower dietary quality with an elevated caloric intake [2, 3]. As a byproduct of the processed foods, a Western diet is also very high in saturated fats [3, 5]. Due to the difference between diets, there exists an array of potential health benefits when transitioning from a Western diet to a MD, and thereby fulfilling daily macro- and micronutrient needs more adequately [3, 6]. Regarding macronutrient distributions, a MD is lower in saturated fats but higher in monounsaturated fats [2]. As for micronutrient discrepancies, a MD is higher in fiber and antioxidants [2]. In addition, the MD has a lower environmental impact compared to a Western diet due to the reduced consumption of animal products and processed foods [2, 6].

There has been increasing incidence of Erectile Dysfunction (ED), hypogonadism, Benign Prostatic Hyperplasia (BPH), Lower Urinary Tract symptoms (LUTs), Urinary Incontinence (UI), and nephrolithiasis evaluated and treated by urologists. Patients with these conditions simultaneously may have associated medical comorbidities such as obesity, hyperlipidemia, hypertension, Diabetes Mellitus (DM), and heart disease. With the rate of obesity in the United States estimated near 40% and demonstrating an increasing trend, prevention approaches have become paramount [7]. Moreover, the incidence of anxiety, stress, and depression has been significantly rising since the covid pandemic [8]. Given the frequency of comorbidities for patients presenting with varied urologic conditions, management approaches ought to consider alternative strategies to aid in mitigating the burden of disease.

The MD is associated with a wide range of health advantages, encompassing maintenance of healthier body weight, diminished occurrences of obesity and metabolic syndrome, lowered risk of Type 2 DM, defensive mechanisms against cardiovascular ailments, and notably decreased susceptibility to cancers, including those affecting the stomach, colorectum, pancreas, and liver [1–3, 5, 6]. While these health effects have been validated, the impact of a MD on urologic diseases is yet to be fully explored. We believe that lifestyle modifications including diet, exercise and behavioral changes may help urologic conditions. Through this review, we aim to summarize the current literature involving the effects of a Mediterranean diet on different urologic conditions: sexual dysfunction, lower urinary tract symptoms, stone disease, and urologic cancers.

Male sexual dysfunction was reviewed in the context of ED, the inability to attain or maintain a penile erection sufficient for sexual performance [9], while female sexual dysfunction was considered personal distress in one or more of the following areas during intercourse: desire, arousal, orgasm, or pain [10]. Lower urinary tract symptoms were reviewed in the context of BPH, the proliferation of smooth muscle and epithelial cells within the prostatic transition zone leading to urinary difficulties [11], urinary incontinence as the involuntary urethral leakage of urine, and nonneurogenic overactive bladder symptoms defined as the presence of urinary urgency, usually accompanied by frequency and nocturia in the absence of a urinary tract infection or other obvious pathology [12]. Stone disease refers to the formation of renal calculi specifically, though stones may form de novo anywhere within the urinary tract. The urologic cancers reviewed included those of primary origin from the prostate, bladder, or kidney.

## Methods

### Search strategy

PubMed and Embase were utilized for this review. The following search terms were used in both search engines: “Mediterranean diet” or “nutrition” or “diet” or “fad diet” or “Paleo diet” or “weight loss diet” or “vegetarian diet” in conjunction with either “Erectile Dysfunction” or “Female Sexual Dysfunction” or “BPH” or “Urinary Incontinence” or “Kidney Stone” or “Over Active Bladder” or “Prostate Cancer” or “Bladder Cancer” or “Renal Cancer”. These searches gave a total of 955 papers.

### Inclusion and exclusion criteria

**Fig. 1:**
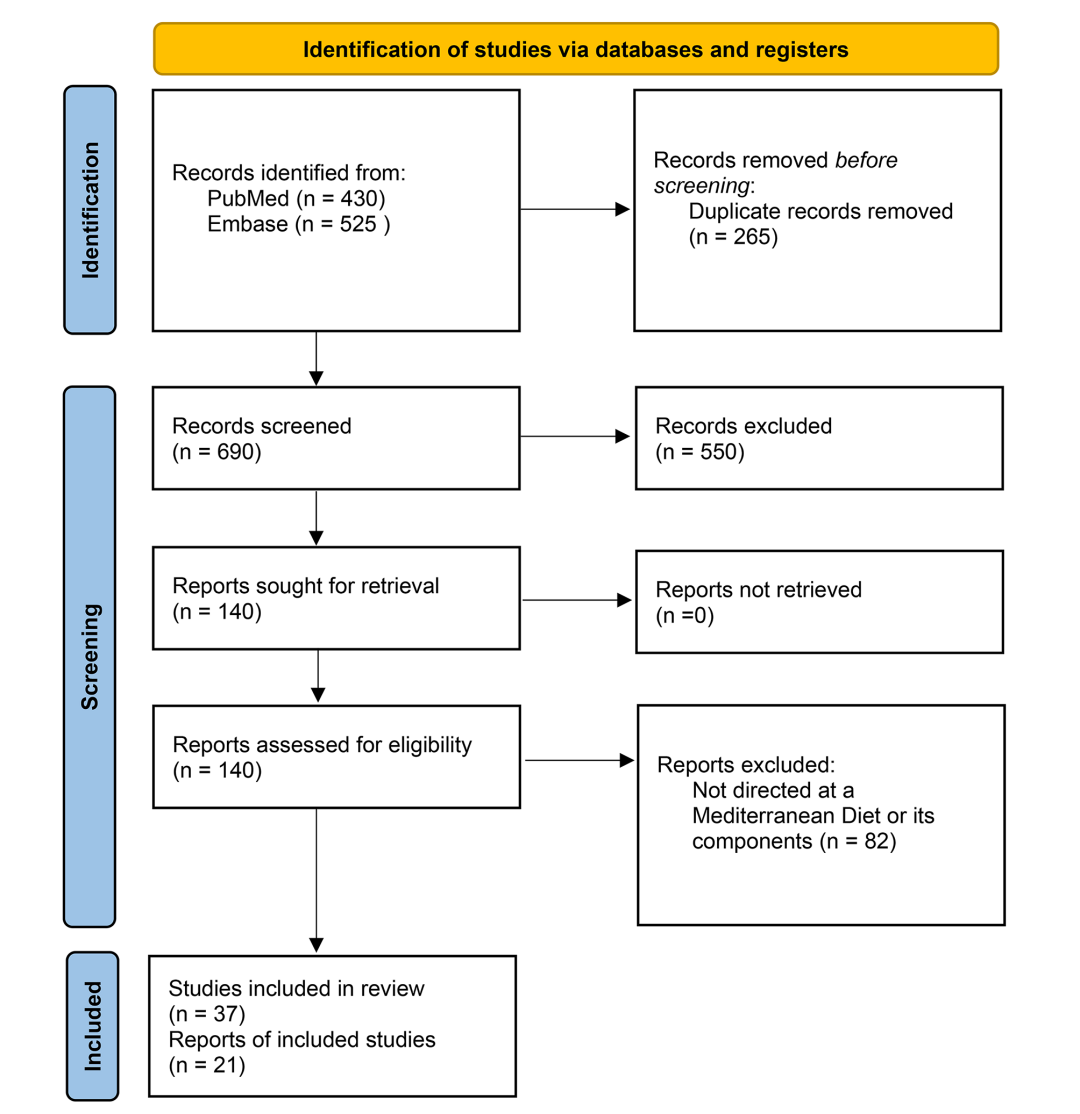
PRISMA Flow diagram for the review of the impact of a Mediterranean Diet on Urologic Disease. Page MJ, McKenzie JE, Bossuyt PM, Boutron I, Hoffmann TC, Mulrow CD, et al. The PRISMA 2020 statement: an updated guideline for reporting systematic reviews. BMJ 2021;372:n71. doi: 10.1136/bmj.n71 [13]

Following the compilation of diverse search results from distinct databases (PubMed and Embase), a precise screening process was enacted by two independent reviewers and discrepancies were resolved the guarantor of this review. 265 records were excluded due to duplication in both databases and 550 records were excluded on title or abstract review only. Inclusion criteria encompassed a spectrum of study designs, specifically randomized controlled trials, cohort studies, cross-sectional studies, and reviews, and meta-analyses. These studies scrutinized the ramifications of a MD or its constituent components on a urologic disease of interest. Notably, studies that diverged from the research focus, case studies, abstracts, editorials, and studies of inadequate quality were systematically excluded. In totality, 58 studies emerged as meeting the defined criteria. A graphical representation of this screening process is visually outlined in Fig. 1.

### Risk of bias

All primary observational studies were assessed for the risk of bias using the Newcastle-Ottawa Scale by two independent reviewers. 11 primary studies were identified to assess the role of a MD on sexual dysfunction, 9 primary studies regarding urinary symptoms, 8 primary studies regarding stone disease, and 9 primary studies regarding urologic cancers. Supplemental Tables 1–4 correspond to the Newcastle Ottawa scoring for primary studies regarding sexual dysfunction, urinary symptoms, stone disease, and urologic cancers respectively. A modified template was instituted for cross-sectional studies by considering the presence of the independent variable for comparison as a case and the lack thereof as a control. All the primary studies included in this review were considered good/high quality studies corresponding to a score of 6–9.

## Results and discussion

### Sexual dysfunction

#### Male sexual dysfunction

The MD has been reported to demonstrate a beneficial effect on male sexual dysfunction, specifically ED [5, 14]. The MÈDITA trial was a clinical trial measuring the impact of a MD compared to a low fat diet in men with Type 2 DM over an 8 year period, which included assessing patient sexual dysfunction [15]. The outcomes underscored a notable decrease in the prevalence of ED throughout the study duration, accompanied by a hazard ratio (HR) of 0.44 (0.19-1.00, *P* = 0.045) for individuals adopting a MD. Moreover, the study identified a decrease in the development of new ED or the progression of existing ED cases, reflected by a HR of 0.41 (0.21–0.83, *P* = 0.011) [15]. Within the Health Professionals Follow-up Study, a cohort of 21,469 men also reported an inverse relationship between adhering to a MD and the incidence of ED [16]. On a robust multivariable model, those with the strongest adherence to a MD compared to those with the lowest adherence had HRs for developing ED as follows: 0.78 (0.63–0.97, *P* < 0.007) for men under 60, 0.78 (0.69–0.87, *P* < 0.001) for men between 60 and 70, and 0.89 (0.81–0.99, *P* = 0.03) for men over 70 [16]. Their model accounted for patient age, race, smoking status, Body Mass Index (BMI), physical activity, marital status, and the use of 5α-reductase inhibitors as well as other medical comorbidities. However one limitation was that their data was aggregated by mail in questionaries directed at health professionals and may not be generalized to the broader public. While weight loss has been shown to have a beneficial effect on ED, the authors hypothesize that the antioxidant rich foods found in a MD improve endothelial function thus more appropriately regulating blood pressure and mitigating certain etiologies of ED [16]. Other trials have assessed the impact of a MD on ED and found improved International Index of Erectile Function (IIEF) scores and reduced inflammatory markers with MD adherence compared to a control diet [17]. Over the course of 2 years, IIEF scores increased by roughly 3.7 points on a 25 point scale in the intervention group, compared to 0.3 points in the control group (*P* < 0.01), in addition participant’s C-Reactive Protein (CRP), a marker of inflammation, was reduced by 0.9 mg/L within the intervention group (*P* < 0.01) [17]. On multivariate analysis, the authors concluded 64% of the IIEF score variance was attributed to differences in nutrient intake (34% of the variance, *P* = 0.01), CRP (16% of the variance, *P* = 0.03), and endothelial function scores (14% of the variance, *P* = 0.045) [17]. Thus, a MD demonstrates a strong potential for reducing systemic vascular inflammation and improving endothelial function, thereby ameliorating the hallmark vascular dysfunction associated with ED.

Cross sectional studies have also corroborated the positive effect of a MD on ED. A study of 555 men with Type 2 DM which examined the prevalence of ED and MD adherence found a prevalence of 16.5% in the highest tertile of diet adherence compared to 26.4% (*P* = 0.01) in the lowest tertile [18]. However, the diabetic men with the highest MD adherence also appeared to have a lower prevalence of obesity or metabolic syndrome, a better glucose and lipid profile, and a higher level of physical activity, thereby potentially confounding the results. Another study of 440 non-diabetic patients found similar results, specifically noting that the consumption of nuts (> twice per week) and vegetables ($\ge$ once per day) played the largest role in reducing ED prevalence with an Odds Ratio (OR) of 0.41 (0.25–0.67, *P* = 0.009) and 0.47 (0.28–0.77 *P* = 0.012) respectively [19]. Other cross sectional studies have continued to report similar results to the aforementioned studies, noting that higher adherence rates to a MD generally leads to a lower prevalence of ED [5, 14, 20]. In addition, varied study populations have continued to demonstrate the potential comorbidities associated with ED, one cross sectional observational study in men with celiac disease demonstrated IIEF scores to be negatively correlated with BMI (*r*=-0.334, *P* < 0.001), however on a multivariate logistic regression accounting for age, hypertension, and diabetes, BMI was no longer a predictor of sexual dysfunction [21]. Therefore, corroborating the plethora of factors which may synergistically potentiate the development of ED.

In addition to mitigating the impact of ED, diet may also play a role on patient’s testosterone levels and improve fertility. An increase in serum total testosterone has been validated for overweight men adhering to a low-fat or low-calorie diet, presumably related to resulting weight loss causing less frequent aromatization of testosterone. A randomized control trial of 118 overweight and obese men continued either a high protein, low fat diet or a high carbohydrate, low fat diet reported a mean increase in total testosterone of 0.68 nmol/L after 12 weeks of diet intervention (*P* = 0.037) [22]. Though of note, no differential effect appeared attributable to patient diet between treatment groups for the change in testosterone (*P* = 0.670), free testosterone (*P* = 0.630) and SHBG (*P* = 0.508) potentially confounding the utility of a strict MD to raise serum testosterone in lieu of the advantages for weight loss in overweight or obese individuals [22]. To evaluate semen parameters, a cross sectional study of 701 healthy men demonstrated higher saturated fat intake to be inversely related to total sperm count (*P* < 0.02) and sperm concentration (*P* < 0.04), translating into a 38% lower sperm concentration and a 41% lower sperm count between the highest and lowest quartile of saturated fat intake [23]. In addition, the results appeared to have a significant dose-dependent association with the highest decile of saturated fat intake demonstrating a 59% (14-80%) lower sperm concentration and a 65% (25-84%) lower sperm count compared to the lowest decile [23]. Though this study did not gauge the utility of a MD directly, an association may be inferred given its dietary emphasis on unsaturated fat. However this evidence is limited by its cross-sectional study design and confounded by the fact that men who had a high intake of saturated fat are also more likely to have an unhealthy lifestyle.

Guidelines have also recommended a multimodal treatment approach due to the plethora of benefits offered by lifestyle interventions. Per the American Urological Association (AUA) guidelines for ED, clinicians ought to counsel men with comorbidities known to negatively affect erectile function that lifestyle modifications, including changes in diet and increased physical activity may improve erectile function (Moderate Recommendation) [24]. Thus, demonstrating the promise of a multimodal treatment approach.

#### Female sexual dysfunction

Similar to male sexual dysfunction, the literature shows a general improvement in female sexual dysfunction (FSD) incidence and symptoms associated with adherence to a MD [25]. The previously mentioned MÈDITA trial also assessed the effects of a MD on FSD. The results noted a HR of 0.44 (0.19-1.00, *P* = 0.048) and 0.50 (0.25–0.99, *P* = 0.045) for the incidence of new FSD and the risk of worsening FSD respectively for those on a MD compared to a low fat diet [15]. Though one limitation for this trial is its limited generalizability for the broad population given all participants were diabetic at baseline. To methodically assess the degree of FSD in the public, Female Sexual Function scores have assessed sexual desire, arousal, lubrication, orgasm, satisfaction, and pain to gauge the impact of a MD [26]. In a clinical trial spanning two years, which scrutinized the effects of a MD in women with metabolic syndrome, noteworthy findings emerged. Those who diligently adhered to the MD experienced a transformation in their total Female Sexual Function scores, registering a substantial shift of 6.4 ± 3.8 points. In stark contrast, the control group exhibited only a marginal alteration of 0.3 ± 0.7 points (*P* < 0.01), underlining the tangible impact of the diet to promote female sexual well-being [26]. However one potential concern of this trial is that it did not specify which diet the control group followed, only that the intervention group consumed greater quantities of fruits, vegetables, nuts, whole grains, and olive oil [26]. In addition, on multivariate analysis to predict Female Sexual Function scores multiple independent predictors appeared significant including BMI (38% of the variance, *P* = 0.01), nutrient intake (20% of the variance, *P* = 0.02), and serum CRP (12% of the variance, *P* = 0.04) [26]. Given that BMI appeared to have a greater impact on the variance in Female Sexual Function scores compared nutrient alterations, the potential benefit of a MD in FSD appears synergistic to the benefits of maintaining a healthy BMI.

Similar to men, cross sectional studies have reported an inverse relationship with a MD and prevalence of sexual dysfunction [25, 27]. One cross sectional study of 595 women with Type 2 DM found women more adherent to a MD demonstrated a FSD prevalence of 47.6% compared to 57.8% with lower diet adherence (*P* = 0.01) [27]. However, the diabetic women with the highest MD adherence also appeared to have a lower BMI, a higher level of physical activity, and a lower prevalence of depression potentially confounding the results and limiting the studies generalizability. Mechanistically, it remains unclear specifically how the diet benefits FSD, however a reduction in general inflammation, as demonstrated by reduced CRP levels, has been hypothesized to play a role in conjunction with increased antioxidant intake [26, 27]. In addition, female sexual dysfunction appears multifactorial and impacted by psychological and physiologic disturbances. For example, in women with burning mouth syndrome, a condition associated with psychological factors such as anxiety, depression, and sleep disturbances has demonstrated significantly negative impact on sexual desire in women [28]. Thus showing that though diet may play a role in improving female sexual dysfunction, a multidisciplinary approach remains necessary. Overall, the benefit of a MD for sexual dysfunction appears to offer a synergistic benefit with allowing patients to maintain a healthy body weight, limiting saturated fat intake, and reducing the inflammatory stress burden.

### Urinary symptoms

#### LUTS and BPH

Although there is a scarcity of scientific literature examining the impact of a MD on LUTS and BPH, certain studies do propose potential associated benefits [29, 30]. Within the Health Professionals Follow-Up Study, a prospective cohort of 32,265 men were queried to ascertain the effects of fruit and vegetable consumption on BPH. The results noted increased vegetable, ß-carotene, and lutein consumption to be associated with reduced incidence of LUTS and BPH as well as improved symptoms [29]. The quintile with the most vegetable, ß-carotene, and lutein intake compared to the least demonstrated an OR of 0.89 (0.80–0.99, *P* = 0.03), 0.87 (0.78–0.96, *P* = 0.004), and 0.83 (0.75–0.92, *P* = 0.0004) respectively for the development of BPH [29]. As the MD remains high in vegetables and leafy greens, such as kale and spinach, which contain high quantities of ß-carotene and lutein, the results corroborate a hypothetical utility for a MD to reduce the incidence of BPH [29]. Though a key consideration of this large analysis was that men with an AUA symptom score of 8–14, consistent with moderate disease, were excluded from the analysis, limiting the insight of a MD for patients with intermediate BPH.

In the Prostate Cancer Prevention Trial placebo-arm participants, 4,770 men who were free of BPH at baseline, were followed over 7 years. Participants with the highest and lowest quintile of total fat consumption were compared to reveal an increased risk for BPH by 31% (*P* = 0.018), daily consumption of red meat compared to weekly consumption was associated with a 38% elevated risk (*P* = 0.044), while vegetable consumption on 4 or more days per week compared to less than once per week decreased the risk for BPH development by 32% (*P* = 0.011) [30]. However, multivariate models that accounted for differences in the type of fat consumed found no association to the risk of BPH. Interestingly, their results also demonstrated that the risk for developing BPH was 27% higher (*P* = 0.025) for those in the highest quintile of polyunsaturated fat intake, 15% lower for the highest quintile of protein intake (*P* = 0.134), and that consuming two or more alcoholic drinks per day compared to one drink or less was associated with a 33% risk reduction for BPH (*P* < 0.001) [30]. Thus, again suggesting a MD may be effective to reduce the risk for LUTS and BPH by virtue of increased consumption of fish, poultry, and leafy vegetables over red meat given elevated protein intake appeared to offer a protective role while daily consumption of red meat appeared as a potential risk factor. Overall, conclusions regarding the impact of a MD to manage BPH remain limited, however maintaining a healthy diet with adequate vegetable and protein intake but low in total fat may limit the risk for developing BPH.

#### Incontinence

Much like the scenario with LUTS and BPH, the available literature concerning the impact of a MD on UI is rather limited. Numerous studies have predominantly focused on the outcomes of weight loss attributable to dietary modifications, rather than specifically addressing nutritional implications on incontinence. While the acknowledged advantages of weight loss for alleviating UI are widely recognized, only a handful of studies suggest an augmentative advantage stemming from dietary factors [31]. Within the Health Professionals Follow-Up Study, a prospective cohort study of 2,960 men were assessed to determine the impact of a MD on incontinence in men with prostate cancer. The results demonstrated an improvement in UI after cancer diagnosis for men with higher vegetable intake (*P* = 0.003) [32]. In addition, men with the highest quintile of polyunsaturated and monounsaturated fat consumption after prostate cancer diagnosis had worsened UI compared to the lowest quintile of consumers (*P* = 0.04 and *P* = 0.02 respectively) [32].However the overall MD Scores were not associated with UI Scores. Though this trial is certainly limited by the fact that all men carried a diagnosis of prostate cancer and the authors did not perform subgroup analysis based on the treatment modality as it relates to UI. While the overall MD Score may not been associated with UI, components of a MD such as high vegetable and unsaturated fat intake appears to offer some advantage in this specific population. Furthermore, dietary implications do not appear exclusive to men.

A cross sectional study investigating the relationship between diet and UI in women similarly found a positive correlation between the ingested saturated fat to polyunsaturated fat ratio and UI; the quintile of patients with the highest ingested saturated fat to polyunsaturated fat ratio demonstrated increased incidence of UI compared to the lowest quintile with an OR of 2.48 (1.22–5.06, *P* < 0.0001) in addition to women with the highest total energy intake demonstrating an OR of 2.86 (1.56–5.23, *P* = 0.007) for reporting UI and this association remained significant across all categories of waist circumference [31]. However, the study reported no significant association between the particular intake of either carbohydrates, protein, or total fat and urinary incontinence [31]. In addition, the study failed to distinguish between stress, urge, or mixed incontinence. Given the cross-sectional study design, these results show that there are benefits from dietary changes to improve or decrease risk of incontinence, irrelevant to weight loss. Another prospective longitudinal study of 5816 women over the age of 40 positively correlated increased total fat, saturated fatty acid, and monounsaturated fatty acid intake to an increased risk for developing stress UI [33]. The quintile of subjects with the highest total fat and saturated fat intake compared to the lowest quintile demonstrated an OR of 2.02 (1.33–3.05, *P* = 0.02) and 2.02 (1.35–3.03, *P* = 0.001) respectively for development of stress UI [33]. Interestingly, an increase in consumption of carbohydrates and sugars was associated with a reduced risk of developing stress UI (*P* = 0.05 and *P* = 0.04 respectively) while no association could be drawn regarding the consumption of starches, however these results lose significance after adjusting for BMI [33]. As overweight individuals are more likely to receive a higher percentage of their energy intake from fat, BMI may be potential confounder for the relationship between high fat intake and the risk for stress UI, nonetheless both BMI and high fat intake remained significant on multivariate analysis, therefore supporting an independent association for stress UI and offering utility in a MD.

Wholistically, diets prone to placing the body in an inflammatory state, such as the Western diet, have been associated with urgency UI. Data from the cross sectional National Health and Nutritional Examination Surveys composed of an analysis of 13,441 women between 20 and 65 years old. Participants were stratified patients into quartiles using a dietary inflammatory index and results revealed the quartile of patients with a most proinflammatory diet had an OR of 1.24 (1.07–1.44, *P* = 0.004) for developing urgency compared to the quartile with the least inflammatory diet [34]. However on a multivariable regression model including all potential covariates, the association between the dietary inflammatory index and urgency UI was attenuated to an OR of 1.04 (1.01–1.06, *P* = 0.017), limiting the utility an alternative diet [34]. Overall, limited studies exist to directly investigate the effects of the MD on incontinence, however the mentioned nutritional assessments do suggest that a MD may have a positive impact on patients with both stress and urgency UI given the reduced quantity of saturated fats as well as high vegetable and antioxidant content.

#### Overactive bladder

Only partial evidence exists regarding the impact of a MD, or diet in general, on overactive bladder (OAB) symptoms and results have demonstrated mixed outcomes [35]. One recently published cross sectional study assessing the relationship between a MD and OAB on 326 patients reported a negative correlation between diet adherence and symptoms (*r*=-0.359, *P* < 0.001) [36]. However of note, the authors also reported a significant correlation between BMI and OAB symptoms (*r* = 0.175, *P* = 0.002) and no significant correlation between BMI and MD adherence (*r*=-0.042, *P* = 0.452), therefore potentially confounding their results [36]. A prospective study of 4,887 men over 40 years old followed for one year determined no association between intake of a particular food group and OAB onset [37]. Interestingly, in men who had no symptoms at baseline, daily intake of beer appeared to offer a protective effect for the onset of OAB demonstrating an OR of 0.38 (0.24–0.61, *P* < 0.001) compared to those who never drink beer, and therefore conflicting with standard practice recommendations for patients with OAB to avoid bladder irritants such as alcohol [37]. However, those who were physically unable to participate in exercise were more likely to develop OAB symptoms with an OR of 2.27 (1.45–3.56, *P* < 0.003) compared to participants who seldom exercise, thus potentially associating OAB onset with a disability status [37]. Though this evidence certainly questions the utility of a MD as a tool to assist with OAB, other studies have reported a relationship between particular nutrient deficiencies, such as vitamin D and potassium, with symptom development [38]. A prospective study of 5,816 women over 40 years of age reported a protective relationship with increased intake of Vitamin D, protein, and potassium to signify a decreased likelihood for OAB onset (*P* = 0.008, *P* = 0.03, and *P* = 0.05 respectively) [38]. The authors hypothesized that a deficiency in Vitamin D might have a correlation with the manifestation of OAB symptoms, particularly as low Vitamin D and diminished protein levels portend muscle wasting as a potential etiology for undermined detrusor function, subsequently contributing to the emergence of OAB symptoms [38]. As a result of Vitamin D and protein remaining highly prevalent in fatty fish, which is commonly consumed in a MD, diet adherence may be implicated with improved OAB symptoms, however more research is necessary before ascertaining conclusions. Overall, the evidence for the adherence to a MD in the context in OAB remains limited however there does appear to be benefit for maintaining a well-balanced diet and remaining engaged in physical activity.

### Stone disease

Dietary factors have an established association with stone disease given the plethora of metabolic risk factors within urine which predispose to nephrolithiasis [39]. Elevated Body Mass Index (BMI), a component of metabolic syndrome, has been closely associated with an increased risk for nephrolithiasis [40, 41]. In addition, insulin resistance in patients with Type 2 DM is associated with defects in renal ammonium production, leading to more acidic urine, a risk factor for nephrolithiasis, particularly uric acid stone formation [42]. General consensus corroborates that a high level of fruits and vegetables, as found in the MD, are effective for ameliorating risk factors for stone formation [39, 43, 44]. A multi cohort study of 6,077 patients assessing the impact of MD adherence over 3,316,633 person-years found an association to decreased incidence of stone formation, reporting a HR of 0.72 (0.59–0.87, *P* < 0.001) [43]. Interestingly, the protective association of a MD diet to the risk of stone disease remained significant across different age and BMI categories, thus establishing utility for a MD outside the context of limiting the stone risk attributed to obesity or metabolic syndrome. Another prospective cohort study of 16,094 patients followed for about 10 years established similar results, with a HR of 0.64 (0.58–0.87, *P* = 0.01) for developing nephrolithiasis with the highest level of MD adherence in comparison to the lowest level of adherence [44]. Though one limitation of this study remains that the cohort examined was composed of younger individuals affiliated with a single institution, reporting an average age of 35 for participants, constraining its applicability to an older demographics. However, some studies have reported results which diverge from consensus. One cross sectional study investigating dietary consumption in stone formers compared to non-stone formers found no association with overall MD adherence but did report stone formers typically consume less olive oil than non-stone formers [45]. Though this study was performed in the context of patients with a BMI > 25, the results detailed 24.4% of stone formers consumed greater than 4 tablespoons of olive oil per day compared to 42.5% of non-stone formers (*P* = 0.019) [45]. In addition, another cross sectional study in 267 participants with metabolic syndrome demonstrated a MD to be negatively associated with a high calcium urinary crystallization risk (*p* = 0.012) and a marginal reduction in the uric acid crystallization risk (*P* = 0.079) [46] Suggesting that despite a lack of significant findings regarding MD adherence, specific portions of dietary fat content may be associated with a lower risk of stone formation as well as a decreased consumption of red meat may play a protective role for the risk of uric acid stones in patients with metabolic syndrome.

The biochemical mechanism for the association of a MD with decreased risk for nephrolithiasis may be attributed to different dietary characteristics. A high level of fruit intake increases pH, thus promoting urinary alkalization as well as increased urinary citrate and magnesium levels, thus limiting free urinary calcium to protect against stone formation [43, 46]. In addition, increased intake of fruits and vegetables also raises overall fluid intake thus further preventing stone formation [44]. Finally, whole grains found in the diet contain phytate which inhibits calcium oxalate crystallization [43, 46]. Though of clinical relevance, certain nuts and leafy greens such as almonds and spinach contain high levels of oxalate which may increase the risk for nephrolithiasis [47]. As such, stone formers should generally avoid the consumption of these foods by replacing them with variants containing lower oxalate content including walnuts and broccoli [47]. A Dietary Approaches to Stop Hypertension (DASH) diet is very similar to a MD, with a focus on many fruits and vegetables, whole grains, low amounts of saturated fats, and lean animal proteins however a DASH diet recommends for further limiting sodium intake. One study assigned a DASH score to participants of the Health Professionals Follow-up Study and the Nurses’ Health Study demonstrated the relative risk of stone disease to be between 55 and 60% for the highest compared to the lowest quintile of diet adherence [48]. In addition, participants with high DASH adherence also appeared to have higher vitamin C intake, similar to what would be expected in a MD. Though a DASH diet differs from a MD based on sodium intake, they otherwise remain very similar, and a benefit attributed to a MD is likely. However for hypercalciuric stone formers, guidelines advocate limiting sodium intake in conjunction with low animal protein consumption [49]. Evidence for these dietary recommendations was concluded based on a randomized five year trial of hypercalciuric men randomized to a low calcium diet or a low salt and protein diet with normal dietary calcium intake [50]. Results highlighted that though both dietary approaches limited urinary calcium levels, however men on a low calcium diet had higher urinary oxalate excretion, a risk factor for stones [50]. Overall, the evidence for a MD to limit the risk for stone disease is robust based on guidelines for the medical management of stone disease, the low sodium alternative DASH diet may prove more worthwhile for hypercalciuric patients.

### Urologic cancers

#### Prostate cancer

Diet has been closely studied in relation to prostate cancer, both as a risk factor and prognostically after diagnosis. In 2005, a sentinel manuscript published by UCSF reported intensive lifestyle changes may impact the progression of prostate cancer [51]. Patients with less than Gleason Grade 7 prostate cancer were assigned to a diet of predominantly fruits, vegetables, whole grains, and soy in addition to 30 min of moderate aerobic exercise for 6 days a week and followed for 12 months. The mean Prostate Specific Antigen (PSA) for patients on active surveillance in the experimental group decreased from 6.23 to 5.98 but increased from 6.36 to 6.74 within the control group (*p* = 0.016), therefore demonstrating benefit within the cohort of patients continuing active surveillance [51]. Though one potential confounder in their analysis is predicated on the measure of PSA kinetics as a surrogate to prostate cancer progression, as intensive lifestyle management may curtail serum PSA for reasons other than limiting prostate cancer. However, the current literature regarding the specific utility of a MD remains ill defined.

The Health Professionals Follow-up Study prospective cohort of 47,867 men found no association between a MD and risk of advanced prostate cancer [52]. However, after assessing 4,538 men diagnosed with non-metastatic prostate cancer, those with the highest diet adherence demonstrated a reduced risk of mortality after diagnosis with a HR of 0.78 (0.67–0.90, *P* = 0.0007) compared to those with the lowest diet adherence [52]. However, the men with the highest MD adherence also demonstrated a lower BMI, less smoking, and more exercise, potentially corroborating the benefit of a healthy lifestyle in the organ confined cancer setting. A cross sectional study comparing aggressive prostate cancer cases discerned a protective association attributed to a MD by demonstrating an OR of 0.66 (0.46–0.95, *P* = 0.007) for the prevalence of highly aggressive prostate cancer between the highest and lowest diet adherence groups [53]. The benefit for a MD within prostate cancer appears related to olive oil to portend a protective effect on mortality after prostate cancer diagnosis. An analysis of the Health Professionals Follow-up Study reported a HR of 0.69 (0.55–0.88, *P* < 0.05) for men consuming 5 or more servings of olive oil per week compared to men who consume no olive oil [52]. Alongside olive oil, other studies have determined compounds such as vitamin E, vitamin C, selenium, and lycopene to potentially have similar benefits [54, 55]. However, the SELECT trial, a prospective analysis of 35,533 men found no significant risk reduction for the combined use of selenium and vitamin E supplementation, rather the cohort with increased vitamin E intake displayed a higher risk for developing prostate cancer compared to placebo [56]. Further, a case control study including 2,590 participants with 1482 incident prostate cancer patients concluded no statistically significant association between a MD and prostate cancer risk with an OR of 0.98 (0.77–1.24) for participants with high MD adherence [57]. Interestingly, the analysis also demonstrated an inverse association between high intake of red/processed meat and any prostate cancer on multivariate regression modeling with an OR of 0.83 (0.70–0.99, *P* = 0.04) [57]. In addition to primary studies, a pooled metanalysis of 10 manuscripts reporting on 33,451 patients discerned no significant association between a MD adherence and prostate cancer [58]. Thus overall, the current literature regarding the impact of a MD on prostate cancer remains inconclusive and additional studies are required to better understand potential associations however there does appear to be some benefit for a MD in addition to a more healthy lifestyle for men under active surveillance for low-risk disease.

#### Bladder cancer

The utility of a MD on bladder cancer also demonstrates mixed results. The European Prospective Investigation into Cancer and Nutrition (EPIC) study, an investigation of 477,312 participants from 10 European countries, reported a negative association, however insignificant, between diet adherence and bladder cancer risk with a demonstrated HR = 0.84 (0.69–1.03, *P* = 0.107) [59]. However, the study did find a 34% decreased risk of urothelial carcinoma for current smokers (*P* = 0.043), and this was more pronounced among heavy and long-term smokers showing a 49% decreased risk, thus the high antioxidant content of a MD is hypothesized to mitigate free radical DNA damage attributed to smoking [59]. In addition, a multicenter case-control study reported a negative association between MD adherence and bladder cancer risk demonstrating an OR of 0.66 (0.47–0.93, *P* = 0.02) for the highest compared to the lowest classification of diet adherence [60]. Specifically reporting the benefits of legumes, vegetables, and fish to promote an anti-inflammatory effect with decreased postprandial oxidative levels [60]. Though on possible limitation of their analysis was a lack of control for the amount of physical activity among participants, which is known to be a protective factor for bladder cancer risk [61], and could certainly confound the extent of impact attributable to diet as those who maintain a healthy well balanced diet are likely to engage in regular exercise.

To further encapsulate the effects of a MD on bladder cancer data, a pooled metanalysis of 13 prospective cohort studies totaling 646,222 participants reported a reduced bladder cancer risk with high diet adherence and demonstrated a HR of 0.85 (0.77–0.93) [62]. The authors related such improvement as possibly related to increased olive oil intake, with the hypothesis that a high amount of polyphenols act as antioxidants to produce an anti-inflammatory effect thereby reducing bladder cancer risk [2, 62]. Though the literature reveals a promising impact for the MD to curtail bladder cancer risk, additional research remains necessary to delineate deeper associations. Overall, the increased antioxidant intake with a MD appears to diminish the risk of bladder cancer, particularly in the setting of increased oxidative stress by smoking.

#### Renal cancer

There has been limited literature regarding long term effects of a MD on renal cancer risk, though evidence exists to associate obesity with the development of renal cancer, particularly as obesity and insulin resistance places the body under increased inflammatory stress. The chronic overload of lipids observed in obese individuals results in an increased release of inflammatory factors, adipokines, and cytokines within adipose tissue [63]. In addition, insulin resistance in obese individuals leads to hyperinsulinemia which contributes to increasing levels of insulin-like growth factor-1, consequently inducing cellular proliferation and inhibiting apoptosis thus favoring tumor formation [64]. This has been evidenced in the clinical setting as a meta-analysis of 282,137 incident cancer cases demonstrated that for each 5 kg/m^2^ increase in BMI, the relative risk for renal carcinoma increases by 24% in men and 34% in women [65].

Though the association between renal cancer and obesity has been validated by numerous sources, there are limited longitudinal prospective analysis particularly assessing a MD as a prevention strategy for renal cancer. Case-control studies assessing diet in Mediterranean countries have related diets rich in olive oil, whole grains, vegetables, and fish to a potentially reduced renal cancer incidence [66, 67]. In 767 renal cancer cases and 1,534 controls, the quintile of patients with the highest intake of cooked vegetables compared to the least demonstrated an OR of 0.64 (*P* = 0.003) for the development of renal cell carcinoma [67]. Interestingly, the highest quintile of poultry intake among participants demonstrated an OR of 0.74 (0.58–0.94, *P* = 0.008) and the highest quintile of processed meat intake demonstrated an OR of 0.64 (0.45–0.90, *P* = 0.006) for the risk of renal cell carcinoma. Conversely, an elevated risk of renal cancer appeared for participants with the highest quintile of bread intake showing an OR of 1.94 (1.4–2.71, *P* = 0.0002) [67]. However, their results found no association with red meat consumption and renal carcinoma risk [66, 67]. The reported hypothesize embraces unsaturated fatty acids found in olive oil and fish, as well as the antioxidants in vegetables to allow for a reduced renal cancer risk [66–68]. Overall, the available literature suggests a MD may have some benefit for renal cancer prevention, however further research is required to determine the promise of such a diet within the context of renal cancer prevention in comparison to the antineoplastic advantage for diets high in antioxidants.

## Conclusion

The literature shows promising results regarding the relationship between a MD and several urologic diseases. Despite these encouraging findings, it is essential to acknowledge the existing gaps in knowledge that necessitate deeper exploration. Conditions such as premature ejaculation, loss of libido, overactive bladder (OAB), and urologic cancers remain areas that require a more comprehensive investigation. By demonstrating congruent results, this research serves to bolster the dietary recommendations for patients experiencing these urologic diseases. In turn, this can reduce the incidence and severity of illness while improving patients’ overall quality of life therefore calling for an interdisciplinary approach between researchers, dieticians, and clinicians.

In conclusion, this comprehensive review encapsulates a pivotal connection where dietary science converges with urologic health. The Mediterranean diet emerges as a promising protagonist, offering a multifaceted approach to address various urologic conditions. The journey from evidence to application, from research to practice, stands as a testament to the profound impact that informed dietary choices may impact disease processes within the realm of urology.

### Electronic supplementary material

Below is the link to the electronic supplementary material.

Supplementary Material 1

Supplementary Material 2

Supplementary Material 3

Supplementary Material 4

## Data Availability

No datasets were generated or analysed during the current study.

## Abbreviations

MD: Mediterranean Diet
ED: Erectile Dysfunction
BPH: Benign Prostatic Hyperplasia
LUTs: Lower Urinary Tract symptoms
UI: Urinary Incontinence
DM: Diabetes Mellitus
HR: Hazard Ratio
IIEF: International Index of Erectile Function
CRP: C- Reactive Protein
AUA: American Urological Association
OR: Odds Ration
FSD: Female Sexual Dysfunction
OAB: Overactive Bladder
BMI: Body Mass Index
DASH: Dietary Approaches to Stop Hypertension
PSA: Prostate Specific Antigen
