# Supplementary material for: Impact of a Mediterranean diet on prevention and management of urologic diseases
Source: BMC Urol. 2024 Feb 26;24:48. doi: 10.1186/s12894-024-01432-9 (PMC10898175; doi:10.1186/s12894-024-01432-9)
Supplement: Supplementary file 1 — Supplementary Material 1 [file 12894_2024_1432_MOESM1_ESM.pdf]

Supplemental Table 1: Risk of bias assessment for primary studies regarding a Mediterranean diet and sexual dysfunction using the Newcastle Ottawa Scale.

Study	Selection	Comparability	Outcome/ Exposure	Total
Maiorino et al. (2016)[a] [15]	****	**	***	9
Bauer et al. (2020) [b] [16]	**	**	***	7
Esposito et al. (2006)[c] [17]	****	**	***	9
Giugliano et al. (2010)[d] [18]	****	**	***	9
Ramirez et al. (2016)[e] [19]	****	**	**	8
Moran et al. (2016)[f] [22]	****	**	**	8
Jensen et al. (2013)[h] [23]	**	**	**	6
Esposito et al. (2007)[i] [26]	****	**	***	9
Giugliano et al. (2010)[j] [27]	****	**	***	9
Romano et al. (2022)[k] [21]	****	**	**	8
Leuci et al. (2022) [28]	***	*	**	6

[a] Accounted for the change in body weight, HbA_{1c}, and depressive symptoms.
[b] Accounted for Age, Race, Smoking, BMI, history of hypertension or hyperlipidemia, physical activity, antidepressant or antipsychotic medication, benzodiazepine use, α-blocker or 5α-reductase inhibitor use, caloric intake, and marital status.
[c] Accounted for BMI, waist circumference, level of physical activity, endothelial function score, baseline IIEF score, and serum CRP concentration.
[d] Accounted for age, BMI, waist circumference, waist-to-hip ratio, physical activity, smoking, hypertension, diabetic medication, duration of diabetes, HbA_{1c}, and lipid parameters.
[e] Accounted for age, obesity, hypertension, smoking, alcohol consumption, hours of exercise, history of ischemia, total cholesterol, and diet score.
[f] Accounted for age, anthropometric (height, weight, waist circumference body composition, blood pressure, and lipid profile), cardiometabolic, medical history (prior depression, high blood pressure, or high cholesterol) and psychologic variables in addition to androgen or medication use. Participants were matched based on age and BMI.
[h] Accounted for period of abstinence, BMI, alcohol consumption, smoking, cryptorchidism, macronutrient consumption, physical activity, and motility duration between ejaculation to sample analysis.
[i] Accounted for changes in nutrient intake, BMI, waist circumference, physical activity, and plasma CRP concentration.
[j] Accounted for age, menopausal state, presence of depression, BMI, waist circumference, waist-to-hip ratio, physical activity, smoking, hypertension status, metabolic syndrome, diabetic medication use, duration of disease, HbA_{1c}, lipid parameters, and total energy intake.
[k] Accounted for age, age at diagnosis, number of months on a gluten-free diet, the presence of hypertension, diabetes, and BMI.
