# Supplementary material for: Impact of a Mediterranean diet on prevention and management of urologic diseases
Source: BMC Urol. 2024 Feb 26;24:48. doi: 10.1186/s12894-024-01432-9 (PMC10898175; doi:10.1186/s12894-024-01432-9)
Supplement: Supplementary file 2 — Supplementary Material 2 [file 12894_2024_1432_MOESM2_ESM.pdf]

Supplemental Table 2: Risk of bias assessment for primary studies regarding a Mediterranean diet and urinary symptoms using the Newcastle Ottawa Scale.

Study	Selection	Comparability	Outcome/Exposure	Total
Rohrmann et al. (2007)[a] [29]	**	**	**	6
Kristal et al. (2008)[b] [30]	***	**	***	8
Maserejian et al. (2010)[c] [31]	***	**	**	7
Bauer et al. (2018)[d] [32]	**	**	***	7
Dallosso et al. (2004a)[e] [33]	**	**	**	6
Zhang et al. (2021)[f] [34]	**	**	**	6
Bozkurt et al. (2022) [36]	***	*	**	6
Dallosso et al. (2004b)[g] [37]	***	**	**	7
Dallosso et al. (2004c)[h] [38]	***	**	***	8

[a] Accounted for age, race, smoking status, BMI, physical activity, alcohol intake, energy intake, protein intake, and polyunsaturated fatty acid intake.
[b] Accounted for age, race, waist to hip ration, and total energy intake.
[c] Accounted for age, total energy intake, race, waist circumference, vaginal birth, menopause, use of antispasmodics or anticholinergics, cardiac disease, diabetes, asthma, and rheumatism.
[d] Accounted for age, time since prostate cancer treatment, and energy intake. Multilevel models also used clinical stage, grade, primary treatment type, PSA at diagnosis, BMI, smoking, and presence of comorbidities, family history of cancer, walking pace, physical activity, and weightlifting.
[e] Accounted for energy intake, age, BMI, and baseline overactive bladder.
[f] Accounted for age, ethnicity, socioeconomic status, education, cardiovascular risk, menopause, smoking, alcohol use, and physical activity.
[g] Accounted for age, total energy intake, total fluid intake, and physical activity.
[h] Accounted for total energy intake, age, presence of stress urinary incontinence, and BMI.
