# Supplementary material for: Impact of a Mediterranean diet on prevention and management of urologic diseases
Source: BMC Urol. 2024 Feb 26;24:48. doi: 10.1186/s12894-024-01432-9 (PMC10898175; doi:10.1186/s12894-024-01432-9)
Supplement: Supplementary file 3 — Supplementary Material 3 [file 12894_2024_1432_MOESM3_ESM.pdf]

Supplemental Table 3: Risk of bias assessment for primary studies regarding a Mediterranean diet and stone disease using the Newcastle Ottawa Scale.

Study	Selection	Comparability	Outcome/Exposure	Total
Taylor et al. (2005)[a] [40]	***	**	**	7
Abate et al. (2004) [42]	****	*	**	7
Rodriguez et al. (2020)[b] [43]	***	**	**	7
Leone et al. (2017)[c] [44]	***	**	***	8
Soldati et al. (2014)[d] [45]	***	**	**	7
Prieto et al. (2019)[e] [46]	***	**	**	7
Borghi et al. (2002)[f] [50]	***	**	***	8
Taylor et al. (2009)[g] [48]	***	**	**	7

[a] Accounted for age, alcohol intake, thiazide diuretics, calcium supplement use, dietary intake of fluid, animal protein, calcium, magnesium, phosphorous, phytate, potassium, sodium, sucrose, vitamin B_6, vitamin C, and vitamin D in addition to baseline weight.

[b] Accounted for age, weight, height and BMI, hypertension, diabetes, the use of thiazides, intake of calcium, vitamin C, vitamin D, fluids and caffeine.

[c] Accounted for gender, age, marital status, education, working hours, weight, height, smoking, physical activity, and chronic diseases.

[d] Accounted for sex, BMI, total cholesterol, fasting glucose, HDL, triglycerides, and blood pressure.

[e] Accounted for age, sex, type 2 diabetes, total daily energy intake, and BMI.

[f] Accounted for the total number of stones formed previously, and the number of episodes of renal colic in the previous year.

[g] Accounted for age, BMI, total energy intake, use of thiazide diuretics, fluid intake, caffeine, alcohol use, history of hypertension, and history of diabetes.
