# Supplementary material for: Impact of a Mediterranean diet on prevention and management of urologic diseases
Source: BMC Urol. 2024 Feb 26;24:48. doi: 10.1186/s12894-024-01432-9 (PMC10898175; doi:10.1186/s12894-024-01432-9)
Supplement: Supplementary file 4 — Supplementary Material 4 [file 12894_2024_1432_MOESM4_ESM.pdf]

**Supplemental Table 4:** Risk of bias assessment for primary studies regarding a Mediterranean diet and urologic cancers using the Newcastle Ottawa Scale.

| Study | Selection | Comparability | Outcome/Exposure | Total |
|---|---|---|---|---|
| Ornish et al. (2004)[a] [51] | **** | ** | *** | 9 |
| Kenfield et al. (2014)[b] [52] | *** | ** | *** | 8 |
| Schneider et al. (2019)[c] [53] | *** | ** | ** | 7 |
| Klein et al. (2011) [56] | **** | * | ** | 7 |
| Moller et al (2013)[d] [57] | **** | ** | ** | 8 |
| Buckland et al. (2013)[e] [59] | **** | ** | *** | 9 |
| Bravi et al. (2018)[f] [60] | **** | ** | ** | 8 |
| Bosetti et al. (2009) [66] | *** | * | ** | 6 |
| Bravi et al. (2007)[g] [67] | *** | ** | ** | 7 |

[a] Accounted for baseline clinical and behavioral outcomes prior to lifestyle intervention.
[b] Accounted for age, time period, total daily caloric intake, BMI, vigorous physical activity, smoking status, PSA screening, and total olive oil intake.
[c] Accounted for BMI, smoking status, family history of prostate cancer, NSAID use, education, and Charlson's Comorbditiy Index.
[d] Accounted for age, region of residence, education, smoking, BMI, energy intake, physical activity, family history of prostate cancer, and diabetes.
[e] Accounted for sex, age, total energy intake, smoking status, BMI, and European region.
[f] Accounted for sex, age, study center, education, BMI, tobacco smoking, nonalcoholic energy intake, history of cystitis, diabetes, or family history of bladder cancer
[g] Accounted for study center, sex, age, years of education, family history of kidney cancer, smoking, alcohol
